# Treatment Outcomes of AIDS-Associated Kaposi's Sarcoma under a Routine Antiretroviral Therapy Program in Lilongwe, Malawi: Bleomycin/Vincristine Compared to Vincristine Monotherapy

**DOI:** 10.1371/journal.pone.0091020

**Published:** 2014-03-14

**Authors:** Albert A. Mwafongo, Nora E. Rosenberg, Wingston Ng'ambi, Alexandra B. Werner, William M. Garneau, Joe Gumulira, Sam Phiri, Mina C. Hosseinipour

**Affiliations:** 1 University of North Carolina Project, Lilongwe, Malawi; 2 University of North Carolina- Chapel Hill, North Carolina, United States of America; 3 The Lighthouse Trust, Lilongwe, Malawi; Istituto Superiore di Sanità, Italy

## Abstract

**Purpose:**

Despite Kaposi's sarcoma (KS) being the most prevalent AIDS-associated cancer in resource limited settings, optimal treatment options remain unknown. We assessed whether bleomycin/vincristine compared to vincristine monotherapy was associated with improved treatment outcomes for AIDS-associated KS among patients initiating combination antiretroviral therapy (cART) in Malawi.

**Methods:**

All patients initiating cART and chemotherapy for AIDS-related KS were identified from an electronic data system from the HIV Lighthouse Clinic from 2002 to 2011. Treatment responses were compared between patients receiving vincristine monotherapy and vincristine/bleomycin. Binomial regression models were implemented to assess probability of tumor improvement for patients receiving vincristine/bleomycin compared to vincristine monotherapy after a complete cycle of chemotherapy (9–10 months). A chi-squared test was used to compare changes in CD4 count after six months of chemotherapy.

**Results:**

Of 449 patients with AIDS-associated KS on chemotherapy, 94% received vincristine monotherapy and 6% received bleomycin/vincristine. Distribution of treatment outcomes was different: 29% of patients on vincristine experienced tumor improvement compared to 53% of patients on bleomycin/vincristine. Patients receiving bleomycin/vincristine were 2.25 (95% CI: 1.47, 3.44) times as likely to experience tumor improvement as to those on vincristine monotherapy. This value changed little after adjustment for age and baseline CD4 count: 2.46 (95% CI: 1.57, 3.86). Change in CD4 count was similar for patients receiving vincristine monotherapy and bleomycin/vincristine (p = 0.6).

**Conclusion:**

Bleomycin/vincristine for the treatment of AIDS-associated KS was associated with better tumor response compared to vincristine monotherapy without impairing CD4 count recovery. Replication in larger datasets and randomized controlled trials is necessary.

## Introduction

Kaposi's sarcoma (KS) is the most common HIV/AIDS associated malignancy in sub-Saharan Africa [1]. In Malawi, KS accounts for up to 34% of all cancers, the majority of which are among HIV-infected persons [2]. Mortality among AIDS patients with KS is substantially higher than that among AIDS patients without KS [3]. Although combination antiretroviral therapy (cART) alone is associated with improved clinical KS response, especially among those with more limited disease [4], chemotherapeutic agents alone or in different combinations may lead to improved KS treatment outcomes [5].

Optimal chemotherapy regimens for the treatment of AIDS associated KS in the cART era and in resource limited settings remain largely unknown. Various chemotherapeutic agents have been used for the treatment of AIDS associated KS as single agents. Although liposomal anthracyclines, such as liposomal doxorubicin, have shown good tumor response and tolerability, their relatively high cost make them unaffordable for use in sub Saharan Africa [6]. Vincristine, an inexpensive and immune-sparing and chemotherapy agent for KS treatment, has been used in the region with modest KS tumor response [7].

Chemotherapeutic agents administered in combinations such as doxorubicin, vinblastine, etoposide, and bleomycin have also been used for KS treatment. Some of these combination regimens, such as doxorubicin/bleomycin/vincristine (ABV), have been associated with better tumor response than single agents [8]–[9]. However, such combination chemotherapy regimens are associated with severe myelosuppression. In a group of patients who already may be immunosuppressed from HIV disease, further use of an immunosuppressive regimen for KS disease may predispose patients to increased risk of opportunistic infections. Consequently, new low-cost combination regimens must be studied with a view to reducing adverse effects, particularly immunosuppression, while maintaining overall efficacy.

Bleomycin/vincristine is one possible combination regimen for resource-limited settings that has never been assessed in the cART era. Bleomycin/vincristine regimen is associated with the least marrow toxicity and has been shown to be an effective regimen in a patient population that has suboptimal marrow function. Additionally, its relative low cost and ease of preparation and administration makes it an attractive option for KS treatment in resource-limited settings [6], [10]. However, direct comparison between bleomycin/vincristine and vincristine only regimen has not been made for effectiveness and immune response.

Vincristine monotherapy has been the mainstay of KS treatment since the inception of the Malawi cART program. However, given perceived sub-optimal treatment outcomes among this population, the Lighthouse Trust adopted a new chemotherapy program including bleomycin/vincristine as initial therapy for advanced disease. Within this program, we investigate if bleomycin/vincristine combination chemotherapy is associated with improved tumor outcomes compared to vincristine monotherapy among KS patients on cART in Lilongwe, Malawi. We also explore whether CD4 count recovery is impaired when comparing the bleomycin/vincristine regimen and vincristine monotherapy in this same population.

## Methods

### Study setting

The study was conducted at the HIV Lighthouse Clinic at Kamuzu Central Hospital in Lilongwe, Malawi. The clinic has been treating HIV-infected KS patients with vincristine chemotherapy since opening in 2002. Bleomycin became available periodically from 2002–2011, with greater availability after 2009. Its use was restricted to patients with advanced KS disease.

Patients on vincristine monotherapy were scheduled to receive 2 mg of vincristine administered once a week for six weeks, then 2 mg every 2 weeks for 12 weeks, then 2 mg every four weeks for 24 weeks, for a total of 18 doses over 42 weeks. Patients on bleomycin/vincristine were given 15 units/m^2^ of bleomycin and 2 mg of vincristine intravenously every two weeks until the maximum cumulative dose of bleomycin (300 units) was reached, typically at 40 weeks.

All patients with HIV/AIDS-associated KS were in WHO stage IV and eligible for HIV treatment, as per Malawi HIV treatment guidelines [11]. All patients were started with the clinic's standard of care first-line cART regimen which consisted of stavudine/lamuvidine/nevirapine. Initiation of chemotherapy typically coincided with initiation of cART.

### Study population and design

Study participants were all HIV-infected patients with advanced KS initiating treatment for both HIV and KS at Lighthouse clinic from July 2002 to December 2011. These patients were identified through the electronic data system (EDS) used for routine monitoring of HIV/AIDS program. Specifically, we screened the entire database for those patients with KS disease and generated a list of patient identification numbers. From this list we verified eligibility. Patients were included in this analysis if the patient chart could be located, if the KS status was confirmed, and treatment regimen information was available. The analysis was restricted to those patients with advanced KS disease stage because these were the only patients eligible for bleomycin/vincristine when both of these drugs become available. KS disease stage was graded as limited stage (T-0) and advanced stage (T-1), based on the AIDS Clinical Trial Group (ACTG) KS staging criteria [12], [13]

We conducted a retrospective cohort analysis of these patients. The primary comparison of interest was tumor improvement at the completion of their KS treatment, typically at 40–42 weeks. We also compared adherence to the chemotherapy regimen and change in CD4 count at six months from KS treatment initiation.

### Data collection and management

In addition to being routinely monitored through the EDS, all patients undergoing KS treatment have two separate paper charts. The first one is specifically for their KS disease management and describes doses given or missed at each visit as well as KS treatment response. Additional information about KS treatment was completed in a primary master file for the patient used for general management of their HIV disease. Both of these paper charts were completed by trained clinicians. We created a dataset by linking these three sources as identified by a file number.

Baseline characteristics of KS patients including age, sex, and CD4 count were obtained from the clinic EDS. CD4 count at six months was also obtained from the EDS. KS disease stage, treatment response, and number and dates of chemotherapy were obtained from the KS paper chart. The primary master files included additional information including location, size or extent of lesions. An independent reviewer, with extensive training and clinical experience in KS, abstracted the aforementioned variables from the three data sources and created a single dataset linking records with a unique identifier. All data were then analyzed using Stata 12.

### Study outcomes

The primary outcome variable was treatment response. Treatment response was graded as complete, partial, stable, or progressive based on the best available description of the lesions before and after treatment as obtained from the two paper patient charts. Complete response was defined as clearance of all Kaposi's sarcoma lesions for more than one month with no record of new lesions developing. Partial response was defined as a decrease by more than half in the size of initial lesions as reported by the clinician assessment. Stable disease was defined as tumor response with no noticeable increase or decrease in tumor burden or change in functionality. Progressive disease was defined as an increase in the size or number of Kaposi's sarcoma lesions. Patients who died while on treatment for their KS had their treatment outcome classified as dead. Patients who failed to attend up to three consecutive scheduled KS clinic visits or could not be traced were classified as defaulters. In most cases, treatment response was recorded for each patient either in the KS treatment charts or in the master file. However, in some cases, we could not determine the treatment outcome after treatment completion because the result was not indicated in the patient's KS treatment chart or the master file. These outcomes were therefore labeled missing.

For some analyses tumor response was dichotomized into improvement or non-improvement. Tumor improvement included complete and partial response. The tumor non-improvement definition varied in the main and sensitivity analyses. In the main analysis, we compared patients with complete or partial response to patients with stable response, progressive response, or death. In this analysis all patients who defaulted or had missing outcomes were excluded. To explore whether those who defaulted or had missing outcomes were biasing the effect estimates, we conducted two additional outcome dichotomizations. In the first we compared patients with a complete or partial response to patients with a stable or progressive response, death, or default. Including defaulters in this way presumed they were more likely to have poor outcomes, something that has been documented in this setting [13]–[15]. Finally, we compared patients with a complete or partial response to all other patients, including defaulters and those with a missing response.

To assess whether chemotherapeutic regimen was associated with impaired CD4 count response, we compared change in CD4 count between the two different regimens after 6 months of cART and chemotherapy treatments.

To document adherence to treatment protocol, we calculated the proportion of completed doses per cycle of chemotherapy for each regimen. For patients on vincristine, we used 18 as the expected number of doses. For patients on bleomycin/vincristine, we used 15 as the expected number of doses, even though some patients on this treatment regimen were meant to receive fewer doses due to their larger body surface area which would make them reach the maximum tolerable dose earlier. However, this was a rare occurrence, 3.6% of patients on this regimen. The total number of doses received was expressed as a proportion of the expected number of doses.

### Data analysis

Descriptive statistics were calculated based on treatment regimens at the time of chemotherapy initiation. We used a Fisher's exact test to compare proportions between the two treatment regimens. We then compared tumor improvement measured at the end of chemotherapy (40–42 weeks). The distribution of the six possible treatment responses was compared: complete, partial, stable, or progressive response, death and default/lost to follow up. Two secondary outcomes were also compared at 6 months: change in CD4 count and proportion of chemotherapy doses taken. Proportions of participants in each category were evaluated using Fisher's Exact tests.

Unadjusted and adjusted regression analyses were then conducted by comparing vincristine/bleomycin and vincristine monotherapy on the probability of tumor improvement at the end of chemotherapy. In adjusted and unadjusted regression analyses, we fitted a generalized linear model to the data using a log link and binomial distribution. The covariates adjusted for in multivariate analysis were age and baseline CD4 count.

### Ethical approval

The use of Lighthouse data for these analyses was approved by the Malawi National Health Science and Research Committee and the School of Medicine Institutional Review Board at the University of North Carolina at Chapel Hill Protection of Human Subjects committee. In addition, all patient records that were used in this study were anonymised and de-identified prior to analysis.

## Results

In the EDS, there were 1061 patient file numbers that indicated the presence of KS. Some (N = 88, 8%) had no evidence of KS after chart reviews and were excluded. For many others (N = 420, 40%) patient characteristics in the EDS, such as name, age, and gender did not match patient characteristics in the patient master file. These discrepancies were due to recycling of patient numbers. When a patient died or transferred out, their number was reassigned to another patient. These patients were also excluded as we did not have sufficient treatment information for analysis. Of the 1061 patient files, 553 (52%) were verified as having KS in the patient master file. Of the 553 patients, some had indeterminate or T-0 KS stage at baseline and were excluded. There were 449 patients (81%) with T-1 stage KS included in the analysis.

Of these 449 patients, 94% received vincristine monotherapy and 6% received bleomycin/vincristine during the period under study. The patient population was predominantly male (64%) and less than 40 years of age (73%) [Table 1]. Patients receiving vincristine monotherapy were more likely to have a CD4 count of more than 200 cells/μL than patients on bleomycin/vincristine (48% versus 19%, p = 0.04). Patients presenting for care in earlier years were more likely to receive vincristine only as bleomycin was not routinely available until later in the study period.

**Table 1:** Baseline characteristics.

Characteristics	Vincristine (N = 421)		Vincristine/Bleomycin (N = 28)		Fisher's Exact Test p-value
Sex					
Female	151	36%	11	39%	
Male	270	64%	17	61%	0.7
Age (Years)					
< = 29	111	26%	7	25%	
30–39	201	48%	11	39%	
40–49	75	18%	8	29%	
> = 50	34	8%	2	7%	0.6
Baseline CD4 (Cells/μL)					
<200	147	53%	13	81%	0.04
>200	133	48%	3	19%	
missing	141		12		
Year of ART initiation					
≤2006	176	42%	3	11%	
2007	54	13%	2	7%	
2008	48	11%	2	7%	
2009	50	12%	4	14%	
2010	52	12%	6	21%	
2011	41	10%	11	39%	<0.001

Overall, the distribution of the six responses was different between the two groups (p<0.001) [Table 2]. The proportion of patients achieving improved tumor response (partial or complete) among those on bleomycin/vincristine was higher than those on vincristine monotherapy (53% versus 29%). Nearly all patients with tumor improvement on both regimens (98%) exhibited only a partial response [Table 2]. A higher proportion of patients on vincristine (25%) experienced tumor progression compared to patients on bleomycin/vincristine (6%), but a higher proportion of deaths was observed among those on bleomycin/vincristine (12%) compared to those on vincristine (1%). Default was more common among those on bleomycin/vincristine (18%) compared to those on vincristine (1%). A missing response in the KS chart was also more common among those on bleomycin/vincristine (39%) compared to those on vincristine (20%).

**Table 2:** Outcomes by treatment type*.

	Vincristine		Vincristine/Bleomycin		Fisher's Exact Test
	N	%	N	%	p-value
Tumor Response					
Complete	2	1%	0	0%	
Partial	93	28%	9	53%	
Stable	149	44%	2	12%	
Progressive	85	25%	1	6%	
Dead	3	1%	2	12%	
Default/Lost	5	1%	3	18%	<0.001
Missing	84		11		
Change in CD4(Cells/μL)					
Decreased	32	17%	0	0%	
Increased 0–100	55	30%	2	50%	
Increased >100	99	53%	2	50%	0.6
Missing	235		24		
Doses Received					
≤50%	120	30%	12	46%	
50–74%	103	26%	3	12%	
75%–99%	42	10%	4	15%	
≥100%	137	34%	7	27%	0.2
Missing	19		2		

*Comparison of populations on vincristine to that on bleomycin/vincristine for tumor response, change in CD4 count, and adherence rates.

There was no notable difference in CD4 count change after 6 months of chemotherapy treatment (p = 0.6) [Table 2]. All patients on bleomycin/vincristine had CD4 count improvement (100%) compared to 83% on vincristine only. However, the majority of patients in both treatment regimens had missing change in CD4 count values.

There were small differences between groups in the proportion adhering to <50% of doses, 50–74% of doses, 75–99% of doses and 100% of doses, with a trend towards better adherence among those on vincristine (p = 0.2). Patients on bleomycin/vincristine were more likely to receive less than half of the expected number of doses than those on vincristine monotherapy regimen (46% versus 30%). And fewer patients on bleomycin/vincristine than on vincristine monotherapy achieved 100% adherence (27% versus 34%).

In the main unadjusted analysis, the probability of tumor improvement was 2.25 times greater among those on bleomycin/vincristine than among those on vincristine alone (95% CI: 1.47–3.44) (Table 3). After adjustment for age and baseline CD4 count, the probability of tumor improvement was 2.46 times greater (95% CI: 1.57–3.86).

**Table 3:** A Comparison of Bleomycin/Vincristine versus Vincristine Monotherapy on the Probability of Tumor Improvement at 40–42 Weeks*.

		Risk Ratio	95% Confidence Interval
**A. Comparison of Tumor Improvement versus non-improvement (stable, progressive) (N = 346)**			
Unadjusted		2.25	(1.47, 3.44)
Adjusted		2.46	(1.57, 3.86)
**B. Comparison of tumor improvement versus non-improvement (stable, progressive, dead, default) (N = 354)**			
Unadjusted		1.88	(1.16, 3.03)
Adjusted		2.06	(1.24, 3.41)
**C. Comparison of tumor improvement versus non-improvement (stable, progressive, dead, default, missing) (N = 449)**			
Unadjusted		1.42	(0.81, 2.51)
Adjusted		1.48	(0.85, 2.60)

*Comparison of the probability of tumor improvement versus tumor non-improvement among patients on Bleomycin/Vincristine versus those on Vincristine monotherapy. In model (A), only patients with recorded tumor information are included. In model (B), all patients who were dead or lost/defaulted were also included and categorized with tumor non-improvement. In model C, all patients who were dead, lost/defaulted, or missing were classified as having tumor non-improvement. All adjusted models control for age and baseline CD4 count.

In the above models, tumor non-improvement was limited to those with stable or progressive KS disease, or death and those who defaulted or missing was excluded. When tumor non-improvement was expanded to include those who defaulted the probability of tumor improvement was 1.88 times greater among those on bleomycin/vincristine compared to those on vincristine monotherapy (95% CI = 1.16–2.03) in adjusted analysis. When tumor non-improvement was further expanded to also include those with missing responses the probability of tumor improvement was 1.48 times greater among those on bleomycin/vincristine compared to those on vincristine monotherapy (95% CI = 0.85–2.60) in adjusted analysis.

## Discussion

Among patients with AIDS-associated KS, the probability of tumor improvement over a 40–42 week period was more than two times higher among those treated with bleomycin/vincristine compared to those treated with vincristine monotherapy. Fifty three percent of patients on bleomycin/vincristine experienced tumor improvement, compared to only 29% of those on vincristine monotherapy. This greater probability of tumor improvement was seen without any noted impairment in CD4 count recovery.

This is the first direct comparison of the effectiveness of bleomycin/vincristine and vincristine monotherapy. In other assessments in sub Saharan Africa and elsewhere, tumor improvement has been estimated at 57%–72% among bleomycin/vincristine patients [16]–[18] and about 60% among vincristine monotherapy patients [19]. Based on indirect comparisons in separate populations [18]–[22], these regimens appeared to have comparable effectiveness. However, our direct comparison in a single population shows that dual therapy is likely more effective. As this observational study was conducted under routine ART program conditions, our findings represent a realistic reflection of effectiveness for AIDS patients with KS and the results may be generalizable to other routine settings in resource-constrained environments.

There is an important consideration with respect to generalizability. We were unable to access patient files for almost all KS patients who had died. This was due to a decision to reassign the patient numbers of these deceased and transferred KS patients to new patients. Consequently, what we measured were treatment responses among a subset of patients with advanced disease who generally survived, rather than the whole population of KS patients. Future research must assess whether dual therapy has benefits on survival in addition to benefits for morbidity.

Due to the observational nature of the work, there were some important differences between the two patient populations at baseline and over time. The patients receiving bleomycin/vincristine were more likely to have lower CD4 counts but had comparable chemotherapy adherence to those patients on vincristine monotherapy. Based on these factors, we would expect tumor improvement to be worse among these sicker, more immunosuppressed patients. Nonetheless, in adjusted analysis accounting for age and baseline CD4 count, those on bleomycin/vincristine were 2.5 times more likely to experience tumor improvement than those on vincristine monotherapy. Although we believe we accounted for important potential confounders, replicating this work in a randomized setting would strengthen the credibility to these findings.

Our sensitivity analyses suggest the primary effect estimate may be exaggerated. In sub-Saharan Africa, death or other adverse outcomes are likely to account for a substantial fraction of cART patients who are lost to follow-up [23]–[25]. When we assumed that all defaulters in this population had adverse outcomes, the probability of tumor improvement was still elevated (RR = 1.9) among those on bleomycin/vincristine compared to those on vincristine alone. When we assumed that both defaulters and patients with missing outcome information had adverse outcomes, the effect estimate still showed a trend in the same direction, though it was less extreme (RR = 1.5). Thus it is possible that this may have biased our primary analysis towards better treatment outcomes in the bleomycin/vincristine group.

CD4 count recovery among patients receiving bleomycin/vincristine was similar to recovery among those receiving vincristine monotherapy after 6 months of chemotherapy, suggesting the dual regimen did not lead to immunosuppression. Since KS is essentially a disease of underlying immunosuppression, it is not surprising that improved KS would occur in tandem with CD4 count improvement [26]. A treatment regimen that shows good tumor response without undue suppression of CD4 count is an ideal option for the treatment of AIDS related KS [27]. However, these findings need to be interpreted cautiously, as the majority of patients in our study did not have CD4 count results at six months. Additional research in a larger population where CD4 count is collected routinely is needed to confirm these preliminary observations.

Chemotherapy adherence rates were suboptimal among patients in both groups, but slightly lower among patients on bleomycin/vincristine than those on vincristine monotherapy. This finding likely reflects two phenomena. First, more frequent shortages of bleomycin rather than patient characteristics may have undermined adherence to the bleomycin/vincristine regimen. Secondly, some larger patients due to their higher body surface area may have completed their full regimen in a shorter period of time, making them appear non-adherent. When “adherent” was categorized as ≥75%, both groups were 42–45% adherent. Given the limitations of our data collection, we could not fully explore all of the different patterns of adherence timing and how they affected the outcome. However, understanding the optimal timing and dosing of bleomycin/vincristine regimen is an important area for future research.

Although a large fraction of patients had tumor improvement, very few (N = 4) had complete tumor improvement. This is in agreement with current literature about the low rate of achieving lasting tumor remission among AIDS associated KS patients [17], [22]. Indeed, AIDS-associated KS is not a curable tumor although durable remission or symptom relief may be reasonable treatment goals.

A number of operational challenges surfaced during the conduct of this research. As noted, many patients lacked CD4 count information, were lost to follow-up, and had inconsistencies in their chemotherapy regimens. These challenges point to structural barriers, such as limited laboratory capacity, inconsistencies in drug availability, and lack of transport to treatment centers. These challenges are common in Malawi, as well as in other parts of sub Saharan Africa [28]–[30] and have impacts on health outcomes. Operational research can help identify bottlenecks and guide implementation to improve delivery of chemotherapy among patients receiving HIV care.

The rapid scale-up of cART over the last decade has led to dramatic improvements in outcomes for HIV-infected patients in resource-limited settings [31], including patients with KS. Additionally, it is known that cART and chemotherapy have treatment benefits over cART alone [32]. In spite of this remarkable progress, there is still a great need for improved management of AIDS-associated conditions, especially KS. Even after cART scale-up, overall one-year mortality rates among KS patients in Malawi are 22% in contrast to approximately 10% among non-KS patients on cART [3]. These discrepancies point to an urgent need for identification of appropriate regimens and effective implementation.

Chemotherapy that effectively treats KS disease with minimal side effects at a reasonable cost is a critical public health need in sub Saharan Africa and other resource-constrained settings. Bleomycin and vincristine administered together were shown to be one such regimen in a routine clinical setting in Malawi. Bleomycin/vincristine produced better treatment outcomes compared to vincristine monotherapy without causing impairment in CD4 count recovery. This finding needs to be confirmed in observational and randomized settings and then considered by policy makers.
